# Safety Listening in High‐Risk Situations: A Qualitative Analysis of Responses to Safety Voice in Aviation

**DOI:** 10.1111/risa.70106

**Published:** 2025-09-14

**Authors:** Alyssa M. Pandolfo, Tom W. Reader, Alex Gillespie

**Affiliations:** ^1^ Department of Psychological and Behavioural Science London School of Economics and Political Science London UK; ^2^ Oslo New University College Oslo Norway

**Keywords:** accident | safety listening | safety voice | team situation awareness

## Abstract

Safety listening—responses to voice acts aimed at preventing harm—can avert organizational failures like airplane crashes. Research often focuses on attitudes and perceptions of listening using self‐report measures; consequently, little is known about how safety listening occurs behaviorally and influences safety outcomes in high‐risk situations. Using directed and summative content analysis, we analyzed 45 transcripts of flightdeck communication before crashes and near misses to develop a framework of safety listening behavior in risky contexts. We also used abductive top‐down theorizing to identify the processes through which such behaviors prevent harm. We propose that effective safety listening behaviors engage with voice through action and sensemaking, whereas ineffective listening behaviors dismiss or exhibit token engagement with speaking‐up. Our analysis illustrates that engaging with voice enables teams to develop shared and accurate situation awareness of emerging risks, thus potentially averting accidents. Our findings demonstrate the importance of a behavioral approach to safety listening, illustrating that assessing listener engagement with safety voice—rather than attitudes—can provide an accurate and practical explanation for how safety listening influences organizational safety outcomes.

## 1 Introduction

Effective safety communication is one of the last lines of defense for preventing organizational accidents (Barling and Frone 2004; Reason 2000). Despite safety communication encompassing both raising safety concerns (“safety voice”; Noort et al. 2019) and responding to them (“safety listening”; Pandolfo et al. 2025), researchers have typically focused on understanding and encouraging safety voice. This focus has yielded six review papers (Krenz and Burtscher 2021; Lainidi et al. 2023; Morrow et al. 2016; Noort et al. 2019; Okuyama et al. 2014; Paul et al. 2025), one meta‐analysis (Tedone et al. 2025), and numerous interventions (for reviews, see Jones et al. 2021 and O'Donovan and McAuliffe 2020). Yet, safety listening—safety voice's counterpart—has received far less attention, despite being equally critical for occupational safety (Pandolfo et al. 2025). Indeed, safety failure investigations show that safety voice often fails when listeners ignore, misunderstand, or dismiss concerns (Hald et al. 2024), and that poor listening contributes to preventable disasters (Table 1).

**TABLE 1:** Example organizational failures exacerbated by poor safety listening.

Case	Year	Voice act	Response	Outcome	Interpretation
Trans World Airlines 514 (National Transportation Safety Board 1974)	1974	The first officer raised concerns about the altimeter, saying, “I hate the altitude jumping around”	The captain did not respond to the concern and changed the subject by saying, “We have a discrepancy in our VORs [Very High Frequency Omni‐directional‐Range; a navigation aid], a little but not much”	The aircraft crashed into a mountain due to flying at an unsafe altitude	Raising concerns is necessary but insufficient; effective responses are required
Challenger space shuttle disaster (Gouran et al. 1986)	1986	Before the shuttle launch, engineers raised concerns about the O‐ring joint seals on the solid rocket booster, saying that their failure could cause a loss of life	Managers understood these concerns but waived them as a “launch constraint” that could be overlooked and had been overlooked in the past with no consequences	The space shuttle broke apart 73 s into the flight, killing seven	Failing to accurately project situations’ future state may lead listeners to underestimate problems
Swissair 111 (McCall and Pruchnicki 2017)	1998	After smelling smoke and declaring an emergency mid‐flight, the first officer radioed the air traffic controller, saying, “We have to land immediate *[sic]*”	The captain replied that he was in the middle of the checklist and “didn't want to be interrupted” so often	The aircraft crashed, with 229 fatalities	Following emergency protocols may be ineffective in previously unencountered situations
Mid Staffordshire hospital scandal (Francis 2013; Reader 2022)	2005–2009	A patient complained about a wound that was seeping	Clinicians dismissed the concerns, despite the wound later being found to have an infected stitch	This example is one of many; 1200 people needlessly died from poor healthcare	Raising concerns is necessary but insufficient; effective responses are required

To prevent organizational accidents, a deeper understanding of how safety listening operates in high‐risk environments is needed. Safety listening is the conduit through which safety concerns must pass to avert harm. Categorizing different safety listening behaviors will clarify how voice leads to accident prevention or occurrence, enabling the development of safety listening assessments and interventions. Likewise, identifying the psychological mechanisms through which safety communications prevent accidents improves models’ predictive accuracy, offering a more nuanced understanding of the conditions in which communication succeeds in averting disasters. Accordingly, we examine how listeners respond to safety voice and how effective safety communication prevents harm.

Although it is listeners’ behavioral responses to voice that shape unfolding situations and contribute to outcomes, most studies use self‐report measures (e.g., surveys) to examine attitudes toward listening, perceptions of feeling heard, and retaliation after speaking up (Pandolfo et al. 2025; Yip and Fisher 2022). We empirically investigate safety listening behaviors by analyzing flightdeck transcripts before incidents. These transcripts capture safety communication in high‐stakes situations, revealing how safety listening behaviors impact safety management and outcomes (Noort et al. 2021b).

This article will develop a framework to observe and evaluate safety listening behaviors in natural contexts and theorize how safety listening behaviors prevent accidents.

### 1.1 Background

In high‐risk industries like aviation, safety communication is crucial for preventing organizational accidents (Barling and Frone 2004). Such accidents result from vulnerabilities in organizations’ defenses, allowing hazards to escalate into disasters (Reason 2000). Thus, effective safety communication may be a final safeguard to halt this escalation. For instance, voicing concerns may interrupt teams’ failing courses of action (Barton and Sutcliffe 2009), thereby enabling sensemaking (Maitlis and Christianson 2014; Weick 1988), prompting corrective action, and strengthening organizational resilience (Curcuruto and Griffin 2023; Flin et al. 2008). Such safety communication, which is central to theories of accident prevention (Bisbey et al. 2021; Salas et al. 2020), comprises safety voice and safety listening.

Safety voice involves individuals speaking up with safety‐related information to prevent harm (Curcuruto et al. 2020; Noort et al. 2019). It improves safety management by building teams’ understandings of risks (Salas et al. 2020), sharing information (Noort et al. 2021c), fostering dialogue on improvements (Curcuruto and Griffin 2023), spurring sensemaking (Blatt et al. 2006), and enabling admitting mistakes (Weiss et al. 2017). Scholars typically conceptualize safety voice as multi‐dimensional, enacted through observable behaviors, and influenced by organizational systems, team norms, and psychological safety (Bazzoli and Curcuruto 2021; Conchie et al. 2012; Nembhard and Edmondson 2006). Safety voice interventions often enhance team communication (Flin et al. 2008) and create voice‐supportive cultures (Edmondson 2018; Jones et al. 2021).

Safety listening—safety voice's counterpart—is “listeners’ behavior responding to safety voice demanding action to prevent harms in organizational contexts” (Pandolfo et al. 2025, 101). Like other forms of workplace listening (Kluger and Itzchakov 2022; Yip and Fisher 2022), safety listening comprises unobservable (e.g., comprehension) and observable behaviors (e.g., paraphrasing, action). Scholars have identified various listening forms—including willful blindness (Cleary and Duke 2019), silencing voicers (Fernando and Prasad 2019), turn‐taking (Itzchakov et al. 2024), and active listening (Jonsdottir and Fridriksdottir 2020)—predominantly conceptualizing effective listening as agreeing with voicers (Pandolfo et al. 2025). This view was likely influenced by the high use of self‐report measures assessing listening attitudes and perceptions of being heard (e.g., Groves et al. 2021; Tucker and Turner 2015). While valuable, these studies do not treat listening as a behavior with tangible consequences. Adopting a behavioral approach is necessary because these behaviors are how listening affects unfolding situations (i.e., they are world‐making; Power et al. 2023). As safety voice requests changes in action (e.g., slowing the airplane), safety listening behaviors are those actions and verbal responses through which action is guided (e.g., maintaining speed or slowing).

For clarity, we use “safety listening” to describe our conceptualization of the broader construct and “safety listening behavior” to refer specifically to our operationalization, namely, observable actions and verbal responses following safety voice.

### 1.2 The Need to Examine Safety Listening and Safety Listening Behaviors

Despite both safety voice and safety listening being essential for accident prevention, researchers have significantly prioritized voice. Compared to the safety voice literature—with multiple systematic reviews and interventions (e.g., Jones et al. 2021; Noort et al. 2019)—a recent conceptual review identified only 57 studies on safety listening (Pandolfo et al. 2025). Likewise, key models routinely emphasize safety voice but neglect safety listening as a necessary antecedent to safety culture and teamwork (e.g., Bisbey et al. 2021; Salas et al. 2020). This focus on safety voice is understandable, as it is the initial act that interrupts unsafe practices (Barton and Sutcliffe 2009), is more visible than the behaviors comprising listening (e.g., comprehension; Kluger and Itzchakov 2022), and because researchers often assume that voice will always be appropriately addressed.

Yet, voicing concerns is futile if listeners dismiss them. Poor responses to complaints have preceded aviation crashes (Noort et al. 2021b), workplace injuries (Tucker and Turner 2015), and institutional failures (Hald et al. 2024); conversely, understanding listeners’ behavior before avoidable failures remains a critical gap in multiple literatures (Pandolfo et al. 2025). Accordingly, this study investigates safety listening behaviors in flightdeck conversations before incidents to develop a framework of safety listening behaviors and theorize the psychological processes through which safety listening prevents harm.

Developing a safety listening behavior framework will improve safety listening's conceptual clarity (Bringmann et al. 2022). In contrast to safety voice's nuanced typologies (e.g., promotive, prohibitive, preventive, hostile; Bazzoli and Curcuruto 2021), effective safety listening is often simplistically conceptualized as addressing the problem, whereas ineffective listening entails ignoring the complaint or retaliating (Moberly 2014). Yet, voice can be misguided and thus must be disregarded. For instance, if a junior doctor voices a wrong diagnosis, effective listening may involve disagreeing; erroneously agreeing would create a misperception and increase risk. Moreover, although scholars often view the safety voice/safety listening/outcome relationship as one‐shot (where an individual speaks up, the receiver determines and enacts their response, and these two conversation turns influence patient care; Long et al. 2020), it may be that a dialogue occurs where teams sense‐make problems, clarify concerns, or correct misunderstandings (Weick 1988). Typologizing different forms of naturalistic safety listening behaviors will clarify what constitutes (in)effective listening and how it prompts further conversation turns.

Understanding how safety listening behaviors manifest and how they affect outcomes will map the mechanisms through which voice reduces harm. Communication theory (Schegloff 1992; Shannon and Weaver 1949) suggests that safety listening prevents accidents by ensuring listeners correctly understand voice messages. Yet, a comprehensive explanation of how listeners engage with various voice messages and their relationship with accidents is lacking. In exploring how safety communications prevent harm in aviation, we theorize the mechanisms by which safety listening leads to safe outcomes in high‐risk contexts.

Finally, deepening understandings of safety listening behavior will enable the development of assessments and interventions to avert future accidents. Taxonomies of observable behaviors that reliably indicate how these behaviors support or compromise performance are needed for developing effective tools and interventions (Flin et al. 2025). Our study sets the groundwork to create these in the context of safety listening in aviation.

### 1.3 Current Study

We analyzed 45 conversations between flightdeck crews, air traffic controllers (ATCs), and other staff before crashes and near misses. Flightdeck conversations reflect actual safety communication in high‐risk situations, illustrating how safety voice and listening manifest and impact safety outcomes (e.g., fatalities, aircraft damage; Noort et al. 2021b). Aviation's standardized roles, linguistic patterns, training, protocols, and risks provide a controlled yet ecologically valid setting to examine these behaviors and their relationship with accidents.

Analyzing flightdeck transcripts grounds safety listening theory in real‐world observations and sharp‐end contexts. Unlike studies using self‐report measures and focusing on listening attitudes, we identify the behavioral manifestations of safety listening. This approach follows recommendations to richly describe phenomena before hypothesis testing and theory building (Bringmann et al. 2022; Rozin 2009). Flightdeck conversations unobtrusively capture naturalistic behaviors during safety incidents and avoid self‐report measures’ limitations (e.g., memory errors, attribution biases). Through this approach, we develop a framework of safety listening behaviors in high‐stakes contexts and conceptualize the mechanisms through which these behaviors prevent harm.

## 2 Methods

We used a comparative case study design (Kaarbo and Beasley 1999) to examine safety listening in flightdeck conversations. This approach systematically compares multiple naturalistic cases to develop generalizable theory. Our UK‐based university ethics committee provided approval, and we wrote this article following the Standards for Reporting Qualitative Research guidelines (O'Brien et al. 2014).

### 2.1 Data

Flightdeck conversations involve dialogues where teams exchange takeoff and landing clearances, share information (e.g., wind speeds), and report issues (e.g., engine failure). English is the standard language (International Civil Aviation Organization 2009); however, local languages are sometimes used for non‐critical communications between cockpit crews and in regions where English is uncommon. Cockpit voice recorders (CVRs) and ATC radio recordings capture flightdeck conversations and are compared in Table 2. These recordings help investigation authorities to understand incidents’ causes, and their transcripts are sometimes included in comprehensive and independently conducted incident reports.

**TABLE 2:** A comparison of cockpit voice recorder and air traffic control radio recordings.

	Cockpit voice recorder	Air traffic control radio recordings
Purpose	Air accident investigation authorities use recordings to understand aviation incidents occurring in their country. After an incident, the authority recovers the recorder and analyzes the recording. After completing investigations, the authority either securely archives the recording or destroys it, depending on their policies. The recordings are overwritten during normal operations	Airports globally record real‐time conversations for operational purposes (e.g., accident investigation, training)
Recorded sounds	All sounds (e.g., warning noises) and conversations, including intra‐ and inter‐cockpit interactions	Conversations and sounds over specific radio frequencies, excluding non‐broadcasted intra‐cockpit conversations
Maximum recording time	Recorders from the 1960s onward typically record the most recent 30 min of audio. Modern recorders (from the early 2000s) record the most recent 120 min. All recorders continuously overwrite previous audio recordings	No maximum limit
Public accessibility	Air accident investigation authorities rarely release full recordings to the public to maintain privacy. They sometimes provide transcripts in incident reports, depending on the country's policy	Air accident investigation authorities sometimes provide these transcripts in incident reports, depending on the country's policy. The public within 15 mi/24 km of airports can access and record these conversations. Volunteers can upload recordings to YouTube for educational purposes like helping student pilots learn aviation jargon

### 2.2 Data Collection

We collected data from two sources (Table 3) and did not require participant consent due to the data's public nature. Following recommendations to select diverse yet comparable cases with outcome variations (Kaarbo and Beasley 1999), we classified incidents into three categories: At least one fatality (142 CVR; 7 ATC), no fatalities but aircraft damage (28 CVR; 648 ATC), and no fatalities and no aircraft damage (360 ATC). Table 4 illustrates our inclusion criteria.

**TABLE 3:** Data source characteristics.

	Noort et al. (2021a)	VASAviation
Description	Academic dataset consolidating incidents from Tailstrike.com, the Aviation Safety Network, and Plane Crash Info	Aviation YouTube channel
Total number of incidents	172	1156 (737 crashes; 419 near misses) as of May 2023, when data collection occurred
Incident type	Fatal and non‐fatal crashes	Fatal crashes, non‐fatal crashes, and near misses
Years	1962–2018	Ongoing, beginning in 2014
Incident location	Worldwide	Worldwide, though many from America
Data type	CVR and ATC transcripts	ATC recordings
Link to data	https://ars.els‐cdn.com/content/image/1‐s2.0‐S2352340921008775‐mmc2.csv	https://www.youtube.com/@VASAviation
Permission granted	By the authors	By the YouTuber (Victor, from VASAviation)

Abbreviations: ATC, air traffic control; CVR, cockpit voice recorder.

**TABLE 4:** Inclusion criteria.

Domain	Include	Exclude
Conversation type	Flightdeck conversations preceding and including an aviation incident	Post‐incident conversations (e.g., search and rescue efforts)
Data type	Cockpit voice recording transcripts Air traffic control radio transcripts Air traffic control recordings	No or partial transcripts or recordings Conversation summaries Duplicates
Language	Transcripts or recordings in English or professionally translated to English	All other languages
Aircraft type	Airplane	Helicopter Hot air balloon
Incident	At least one instance of safety voice At least one safety hazard	No safety voice Status quo and unremarkable flights

We assigned numbers to each incident and sampled from each category using a random number generator. Noort et al. (2021b) found that crew resource management training improved safety listening; to maximize our framework's comprehensiveness, we ensured our sample included cases pre‐ and post‐implementation (around 1981 for large American carriers and later for smaller operators and other countries; Kanki et al. 2019).

We transcribed all recordings and sampled until data saturation (i.e., adding additional transcripts would not give new information). Following Guest et al. (2020)’s recommendations, we first analyzed an initial set of 18 transcripts (six of each category) to establish a base size of information gleaned regarding types of listening behavior and mechanisms for harm prevention. Next, we analyzed three additional transcripts at a time (one of each category), comparing new information in these transcripts with that gained in the initial 18. We stopped after four batches (i.e., 12 transcripts) because we reached our 0% new information threshold. Last, we tested our findings by adding 15 new transcripts. During sampling, we excluded five incidents (two no safety voice, two non‐English, one post‐incident). All authors independently and collectively reviewed transcripts, validating analyses and ensuring data saturation was not prematurely reached.

### 2.3 Data Analysis

**FIGURE 1:**
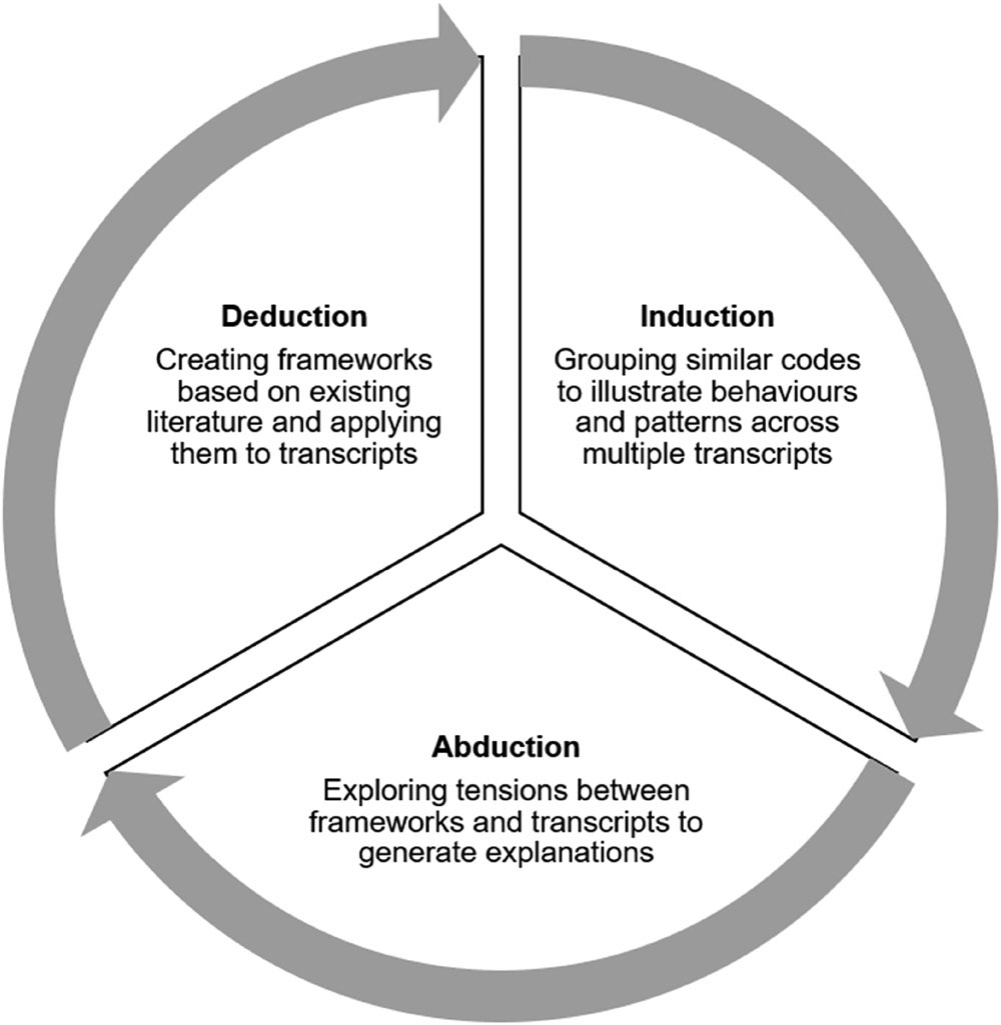
Data analysis process summary.

We used a pragmatist approach, interpreting actual human activity in safety‐critical environments (Gillespie et al. 2024). Figure 1 summarizes our analysis, which moved between deductive, inductive, and abductive frames to address both research objectives.

All authors are research psychologists with qualitative experience. Authors 1 and 2 have consulted on aviation organizational cultures, and Authors 2 and 3 have experience analyzing aviation conversations. Author 1 led data collection and analysis, with all authors regularly discussing case inclusion, data saturation, and finding interpretation.

#### 2.3.1 Developing a Framework of Safety Listening Behaviors

We conducted a content analysis to develop a framework of safety listening behaviors, coding textual data into countable categories (Berelson 1952). We first used a directed approach (coding using deductively generated classifications) and then a summative approach (exploring statements’ nature and contexts inductively; Hsieh and Shannon 2005). Table 5 provides a sample coded transcript.

**TABLE 5:** Example coded transcript for Air Canada 759.

In 2017, Air Canada 759 (“ACA759”) accidentally lined up for landing on taxiway C (containing four aircraft) instead of runway 28R. United Airlines Flight 1 (“UAL1”) communicated this error on the open channel, and the ATC told ACA759 to abort landing				
Line	Speaker	Transcript	Code	Interpretation
1	ACA759	Tower, good evening, Air Canada 759 with you on the Bridge visual 28R		
2	ATC	Tower, Quiet Bridge visual 28R, cleared to land. Wind 270 at 8		
3	ACA759	Cleared to land 28R, Air Canada 759		
4	ACA759	And Tower, just wanna confirm—it is Air Canada 759, we see some lights on the runway there. Please, confirm we are cleared to land?	Muted safety voice	ACA759 said that they saw unexpected lights on the runway
5	ATC	Air Canada 759, confirm. Cleared to land runway 28R. There is no one on 28R but you	Safety listening—Elaborating	ATC did not acknowledge the lights seen by ACA759
6	ACA759	Okay, Air Canada 759	Safety listening—Implementing	ACA759 confirmed the information, resulting in a shared misperception that there was no problem
7	UAL1	Where's this guy going? He's on the taxiway!	Amplifying safety voice	UAL1 told the open channel that ACA759 was landing on taxiway C, revealing the shared misperception
8	ATC	Air Canada, go around	Safety listening—Implementing	The ATC told ACA759 to abort landing
9	ACA759	In the go around, Air Canada 759	Safety listening—Implementing	ACA759 confirmed that they aborted landing, thereby averting the accident
10	ATC	Air Canada 759, looks like you were lined up for CHARLIE there. Fly heading 280, climb and maintain 3000’		
11	ACA759	Heading 280, 3000’; Air Canada 759		
12	UAL1	United 1, Air Canada flew directly over us		
13	ATC	Yeah, I saw that, guys		
14	ATC	Air Canada 759, contact NorCal 135.1; will get you in a couple minutes		
15	ACA759	135.1, Air Canada 759		
16	ATC	United 1, where do they get you going here yet?		
17	UAL1	We're ready!		

Abbreviation: ATC, air traffic controller.

Using directed content analysis, we deductively identified cases of safety voice and listening behaviors. We operationalized safety voice as communication requesting action on hazards, excluding routine safety communications (e.g., ATCs giving take‐off clearance) as they did not involve concerns (Noort et al. 2019). Our operationalization included instances where ATCs issued instructions indicating a problem with the current course of action, attempting to correct behavior and reshape understandings of the situation (consistent with Barton and Sutcliffe 2009). For example, we categorized an ATC instructing a cockpit crew to climb to avoid mountains as safety voice. We operationalized safety listening behaviors as observable responses to safety voice (Pandolfo et al. 2025; see Appendix for our initial coding frame).

After identifying safety voice and listening behaviors, we conducted a summative content analysis to cluster and label them. This inductive analysis involved systematically sampling, comparing, and interpreting sentences and grouping similar behaviors (e.g., listeners hearing safety voice). We organized these clusters in an Excel spreadsheet, iteratively labeled them, noted edge cases, and used abduction to address discrepancies between the deductive framework and the transcripts (Kennedy 2018). We developed superordinate categories encompassing multiple safety listening behaviors (e.g., engagement) and compared these with existing conceptualizations. This process resulted in a safety listening behavior framework, which we verified using the additional 15 transcripts.

#### 2.3.2 Conceptualizing How Safety Listening Behaviors Prevent Harm

Our second analysis investigated how safety listening behavior prevents accidents, adapting guidance for inductive and abductive top‐down theorizing (Sætre and Van De Ven 2021; Shepherd and Sutcliffe 2011). To explore how safety voice and listening behaviors interacted to avert accidents, we analyzed the transcripts using existing concepts (e.g., how listening fosters shared understandings) and abductively developed and refined our conceptualization. We analyzed multiple conversation turns within and across transcripts to identify patterns in how safety communication behaviors impacted outcomes.

Through this analysis, we identified a core mechanism through which safety listening behaviors convert safety voice into meaningful outcomes, specifically by developing team situation awareness. We explored this finding in four steps. First, we observed how listening behaviors contributed to situation awareness and accident prevention, identifying (a)typical manifestations. Second, we examined specific instances to illustrate the process’ various forms. Next, we explored unexpected and contradictory observations, using abduction to refine our theorization. Last, we verified the proposed explanation using the additional 15 transcripts. This process created a conceptualization detailing how safety listening behaviors contribute to harm prevention.

## 3 Results

The data corpus of 45 incidents (available here: https://osf.io/nm7hz/?view_only=0b1a22ab52384728baa24d4c51b8e4af) comprises 6010 lines and 54,121 words. Thirty‐two incidents have official reports, and Table 6 describes incidents’ characteristics. Following Kaarbo and Beasley (1999)’s recommendations, we present our findings as brief narratives alongside coded excerpts and case descriptions.

**TABLE 6:** Incident characteristics.

Outcome	Incident name	Summary	Year	Country registration	Country airspace	Number of lines	Number of words	Recording duration (min)
Fatality	Air France 447^a^	Stalled after icing on pitot tubes and crashed into ocean	2009	France	Brazil	60	1587	125
	Air New Zealand 901^a^	Crashed into mountain	1979	New Zealand	Antarctica	76	814	32
	Asiana Airlines 214^a^,^b^	Collided with a seawall short of the runway	2013	South Korea	The United States	379	3142	125
	Cessna N7022G^a^	Low altitude and flight path deviations resulted in crash on approach	2021	The United States	The United States	59	512	5
	Delta 191^a^	Windshear during approach caused runway undershoot	1985	The United States	The United States	107	791	30
	Eastern Air Lines 401^a^,^b^	Crews were preoccupied with a malfunctioning landing gear indicator light, did not notice the autopilot disconnected, and crashed	1972	The United States	The United States	140	929	9
	Horizon Air^a^	Crashed after airport employee hijacked aircraft and committed suicide	2018	The United States	The United States	40	999	11
	KLM 4805/PanAm 1736^a^	Crashed after KLM initiated takeoff, whereas PanAm was still on the runway	1977	The Netherlands	Spain	114	849	4
	LaMia 2933^a^,^b^	Crashed into mountains during descent	2016	Bolivia	Colombia	54	585	114
	Lot Polish Airlines 5055	Crashed after engine failure and fire	1987	Poland	Poland	38	221	30
	Southwest 1380^a^,^b^	Experienced left engine failure leading to an explosive decompression	2018	The United States	The United States	669	6126	120
	Thai Airways 601^a^	Undershot the runway while landing during a typhoon; crashed into the sea	1967	Thailand	Hong Kong	49	422	13
	United 553^a^	Crashed after stalling in go‐around	1972	The United States	The United States	67	588	7
	US‐Bangla Airlines 211^a^	Crashed on landing due to pilot mental breakdown and loss of situation awareness	2018	Bangladesh	Nepal	166	1768	117
	UPS 1354^a^,^b^	Crashed short of the runway during approach	2013	The United States	The United States	463	3786	93
Aircraft damage, no fatalities	Ameristar 9363^a^,^b^	Overran the departure end of the runway after the captain rejected takeoff; the aircraft's right elevator was jammed	2017	The United States	The United States	801	6403	124
	Cathay Pacific 780^a^,^b^	Encountered a dual engine thrust control problem requiring an emergency landing	2010	Hong Kong	Hong Kong	247	2490	120
	Eastern Air Lines 3452^a^,^b^	Almost overran the runway upon landing	2016	The United States	The United States	68	401	60
	Japan Air Lines 907/Japan Air Lines 958^a^	Near mid‐air collision	2001	Japan	Japan	74	800	15
	Kalitta 808^a^,^b^	Crashed after stalling	1993	The United States	Cuba	32	135	30
	Key Lime 970/N416DJ^a^	Mid‐air collision	2021	The United States	The United States	39	431	3
	Korean Air 2033	Overran runway after a missed approach in bad weather	1994	South Korea	South Korea	20	228	Not given
	South African DC‐4	Mid‐air collision	1962	South Africa	South Africa	50	555	Not given
	Southwest 345^a^,^b^	Nose gear collapsed upon touchdown	2013	The United States	The United States	451	3193	34
	Transair 810^a^	Crashed into ocean following mechanical failures in both engines	2021	The United States	The United States	82	978	8
	Trans‐Air Service 671^a^	Encountered engine separation mid‐flight	1992	Nigeria	France	197	903	26
	United 9963^a^	Take‐off warning horn sounded; crew opted to take off while investigating; the aircraft struck a drainage ditch and caught fire	1968	The United States	The United States	24	132	1
	UPS 1307^a^	In‐flight cargo fire	2006	The United States	The United States	222	1612	31
	US Airways 1549^a^	Aircraft landed in Hudson River after bird strike, which shut down both engines	2009	The United States	The United States	331	3024	30
	WestJet 2425^a^	Tractor backed another aircraft into WestJet 2425 as it was taxiing to the gate	2018	Canada	Canada	66	606	5
No fatalities, no aircraft damage	Aer Lingus 68N/N990FV^a^,^b^	Another aircraft crossed into Aer Lingus’ path during its take‐off roll resulting in a near‐collision	2015	Ireland	Switzerland	64	498	5
	Aer Lingus 123	Right engine failure on takeoff	2021	Ireland	Ireland	41	495	4
	Air Canada 759^a^	Aircraft nearly landed on taxiway with five aircraft	2017	Canada	The United States	15	151	1
	Air Canada Rouge 1633/American Airlines 2172^a^,^b^	The aircraft was cleared for takeoff, and another aircraft was cleared to land at the same time on the same runway	2023	Canada	The United States	44	265	3
	Air China 428	Aircraft nearly collided with mountains	2017	China	Hong Kong	27	191	2
	Caravan N333LD	Passenger with no flying experience landed aircraft with ATC guidance	2022	The United States	The United States	49	586	5
	Delta 1943/American Airlines 106^a^,^b^	The aircraft was takingoff when another aircraft crossed the runway without clearance, resulting in a near‐collision	2023	The United States	The United States	42	335	4
	Diamond N859PA	Military threatened to shoot down aircraft mistakenly trespassing in temporary no‐fly zone	2021	The United States	The United States	38	608	4
	Delta 873	Near runway collision	2016	The United States	The United States	84	939	6
	DHL 1491	Encountered smoke warning indication; returned to airport	2023	Germany	The Netherlands	61	586	6
	FedEx 1432/Southwest 708^a^,^b^	The aircraft descended toward an active runway before breaking off its approach due to departing traffic on the same runway	2023	The United States	The United States	27	254	4
	KLM 1080/Transavia 5193	Near mid‐air collision	2018	The Netherlands	The Netherlands	24	174	2
	Singapore 221	Difficulties landing in bad weather resulted in three aborted landings	2016	Singapore	Australia	42	469	4
	United 1448^b^	Failed to follow taxiing clearance in thick fog and ended up at the edge of the runway it had just landed on	1999	The United States	The United States	247	3371	28
	Virgin Australia 669	Started takeoff on wrong runway	2019	Australia	Australia	20	189	3

Abbreviation: ATC, air traffic controller.

^a^ Has an incident report.

^b^ Incident added for verification.

### 3.1 Developing a Framework of Safety Listening Behaviors

#### 3.1.1 Safety Voice

Before investigating safety listening behaviors, we first examined safety voice. We identified 172 voice acts, which included voicers demanding help, asking listeners’ permission (e.g., to level off), requesting action changes (e.g., to turn left), or seeking clarification (e.g., whether others smelled an odor; Table 7 shows examples). Voicers sometimes engaged in muted voice (e.g., hinting concerns; Table 7, Quote 5). Additionally, voice could be misguided (Hald 2023)—for instance, when an ATC instructed the wrong aircraft to descend (Table 7, Quote 6).

**TABLE 7:** Safety voice examples.

Quote	Interpretation
1. “I've got a serious situation here about my pilot. He's incoherent, no idea how to fly the airplane but I'm maintaining 9,100'” (Passenger, Caravan N333LD, Line 3)	The passenger told the ATC that the situation was no longer business as usual—The pilot was incapacitated, and they did not know how to fly the airplane
2. “Postman 1491, MAYDAY MAYDAY MAYDAY, request level off at 6000'” (Cockpit, DHL 1491, Line 1)	The “maydays” indicated that the situation was no longer business as usual and that instead of continuing to climb after takeoff, DHL 1491 wished to level off
3. “Transavia 5193, left turn immediately heading 180” (ATC, KLM 1080/Transavia 5193, Line 18)	The ATC told Transavia 5193 to turn left to avoid hitting another aircraft
4. “Smells like wood burning. smell that?” (First officer, UPS 1307, Line 15)	The first officer noticed a strange smell and asked others if they also smelled it
5. “Where's [Mount] Erebus in relation to us a[t] the moment?” (First officer, Air New Zealand 901, Line 50)	The first officer hinted their concern about hitting the mountain (muted safety voice)
6. “Japan air niner zero seven, descend and maintain flight level three five zero, begin descent due to traffic” (ATC, Japan Air Lines 907/Japan Air Lines 958, Line 51)	The ATC mistakenly told Flight 907 to descend despite meaning to tell Flight 958 to descend (misguided safety voice)

Abbreviation: ATC, air traffic controller.

#### 3.1.2 Safety Listening Behaviors

**FIGURE 2:**
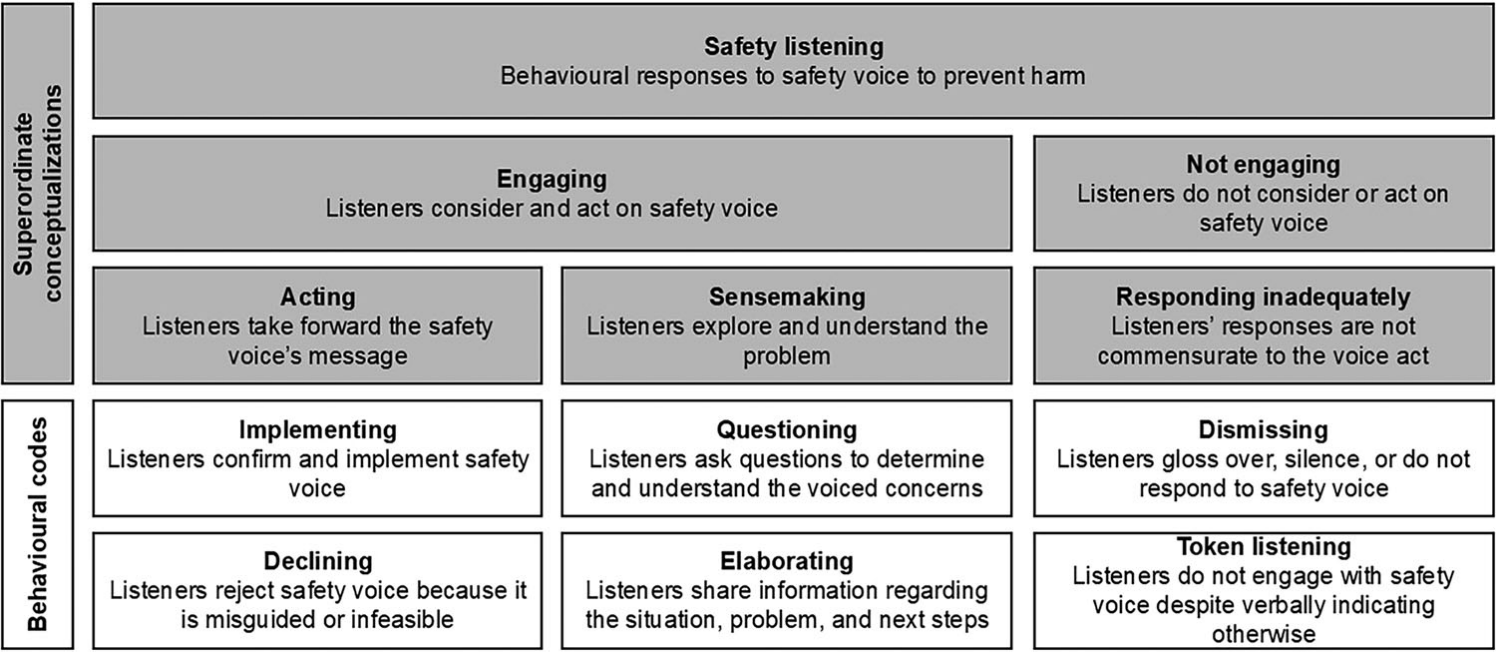
Safety listening taxonomy.

We examined the transcript lines following safety voice, considered them in context, and grouped thematically similar types of safety listening behaviors (Figure 2). We found that effective listening behaviors engage with concerns, where listeners consider the voiced information. Engagement manifests through *action* and *sensemaking*. Conversely, non‐engagement characterizes poor safety listening, where listeners disregard safety voice.

##### 3.1.2.1 Acting

The first type of engagement was action, where listeners indicated that they heard the concern and maintained safety by *implementing* or *declining*.

###### 3.1.2.1.1 Implementing

This common action involved listeners agreeing with and acting on the voicer's concern. For instance, in Virgin Australia 669, the aircraft began taking off from the wrong runway. The ATC intervened saying, “Velocity 669, stop! Stop! You seem to be taking off on [runway] 30 there” and the crew responded “669 stopping” and halted the takeoff.

Listeners also gave permission after voicers suggested a new action. For instance, American Airlines 2172 was cleared to land on the same runway as another aircraft taking off. Noticing the conflict, the American Airlines cockpit announced, “American 2172 going around.” The ATC confirmed this decision by responding, “roger. American 2172, turn right heading 270,” indicating agreement with the proposed missed approach.

Crews occasionally acted on safety voice without verbal acknowledgement. For instance, during a near runway incursion involving Delta 873, the ATC twice mumbled, “Delta 873, cancel take‐off clearance.” Although Delta 873 did not verbally confirm, they demonstrated understanding by halting the takeoff. Another example is when the ATC observed N990FV crossing in front of Aer Lingus 68N during takeoff. The ATC instructed N990FV to “hold position here!” and N990FV complied, preventing a collision.

Voicers also broadcasted safety messages without directing them to specific recipients. When this happened, listeners (typically ATCs) redirected the message to the appropriate party to ensure correct actions were taken. For instance, a tractor was pushing a Sunwing airplane toward a collision with WestJet 2425. The WestJet crew said, “Stop Sunwing,” and the ATC redirected this to the tractor, saying, “Tractor 540, stop there.” Unfortunately, the tractor did not quickly stop, leading to a collision.

####### 3.1.2.1.1.1 Declining

Listeners declined safety voice when they believed voicers were misguided or that requested actions were infeasible. Although researchers often assume that voicers are always correct, we observed infrequent cases where they were mistaken, leading listeners to disagree and refuse to act on wrong information. For instance, in United 9963, a warning horn signaled an incorrect take‐off setting (i.e., the flaps). The captain mistakenly voiced, “It must be the trim,” but the first officer correctly disagreed, saying, “No, it's in the green band now. It can't be the trim.” The crew continued taking off while sensemaking the problem, ultimately contributing to the crash.

Declining also occurred when voicers’ requests were infeasible. For example, in LaMia 2933, the cockpit requested, “Vectors to the runway!” as they were attempting to land with electric and fuel failures. The ATC declined, explaining, “we lost radar signal. I can't see you.”

In Caravan N333LD, after the pilot became incapacitated, a passenger with no piloting experience tried to land the aircraft. The ATC instructed the passenger to input a radio frequency and received no response. They then asked, “did you copy the frequency?” The passenger replied, “No,” indicating they could not follow the instruction. Recognizing this, the ATC provided new instructions, helping the passenger safely land the plane.

##### 3.1.2.2 Sensemaking

The second type of engagement with safety voice is sensemaking. It is distinct from acting as it involves listeners determining whether speaking up occurred, what the concern was, and how to address it.

###### 3.1.2.2.1 Questioning

This form of safety listening behavior involves checking whether colleagues spoke up and clarifying concerns using questions. For instance, listeners requested repetitions or clearer communications (e.g., by fixing radio static) to confirm whether safety voice occurred. For example, in KLM 4805 (took off without clearance and collided with another plane), the flight engineer said, “Is he not clear then?” inquiring if the other aircraft was still on the runway. The captain responded, “What do you say?”, leading the flight engineer to repeat the question. Another example is when Delta 191's first officer remarked, “Lightning coming out of that [cloud]” and the captain responded, “What?”, prompting repetition.

After determining that voice occurred, teams commonly sought clarification on the concern's implications. For instance, in Horizon Air (hijacked airplane), a crew member reported to the ATC, “aircraft just passed off our right, and it's rolling.” The ATC clarified: “What's the aircraft on Runway 16C?”. The crew provided more details: “His wheels are smoking left and right. He's rolling now right down the runway” and “We saw him coming down the cargo area via D.”

Another example is when the Transair 810 crew told the ATC, “We have lost [our] number one engine and we are coming straight to the airport.” The ATC requested further details by asking, “how many individuals are on board?” and “do you have the airport in sight?”.

###### 3.1.2.2.2 Elaborating

Elaborating involved team members interpreting the problem, determining how to address it, and updating each other's understanding of the unfolding situation. For example, UPS 1307's first officer remarked, “smells like wood burning smell that?” The second officer replied, “yeah. I smelled it for a couple of seconds,” adding more information. They then discussed where the smell was coming from (“its *[sic]* more in the back”), what it could be (“does not appear to be any smoke or haze”), and how to address it (“does it seem to get any better with the packs smoke uh checklist?”). They eventually correctly concluded that the problem was cargo smoke and addressed it accordingly.

Another example is Air France 447, where a stall occurred because First Officer‐1 was pulling back on one throttle, whereas First Officer‐2 was pushing forward on another. First Officer‐2 repeatedly said “climb” and First Officer‐1, confused, elaborated, saying, “But I've had the stick back the whole time!” The captain realized the issue, saying, “No, no, no… Don't climb… no, no,” whereas First Officer‐2 said, “Descend, then… Give me the controls.” This communication allowed them to temporarily recover.

##### 3.1.2.3 Not Engaging

Analysis of cases where listeners did not engage with safety voice revealed two behaviors: dismissing and token listening. These behaviors were challenging to identify from single dialogue turns and became apparent when examining subsequent actions and outcomes reported in transcripts and incident reports.

###### 3.1.2.3.1 Dismissing

This response consisted of dismissing and ignoring and involves hearing voice but insufficiently considering its content. Researchers often frame dismissal as overt (e.g., defensiveness)—for example, Kalitta 808, where the flight engineer and first officer warned, “Stall warning” and the captain responded, “I got it, back off.” The airplane stalled and crashed.

Conversely, we often found dismissing to be rare and implicit, manifesting as glossing over and failing to consider concerns. This difference may be due to air crews’ training and the high‐risk context. For instance, in US‐Bangla Airlines 221, the aircraft mistakenly approached Runway 20 despite clearance for Runway 02. The ATC correctly stated, “You are going towards Runway 20,” but the pilot in command dismissed this, incorrectly insisting, “we are going to Runway 02.” The pilot's lack of situation awareness became apparent when he privately asked the first officer, “Can we see the Runway? We have set up everything, all done but we are not seeing the Runway.”

Ignoring is a lack of verbal or behavioral response to safety voice. In Air France 447, ice crystals caused the autopilot to disconnect, resulting in temporary airspeed measurement inconsistencies. Rather than correctly keeping the aircraft at the same altitude, First Officer‐1 kept climbing it, leading to a stall. When First Officer‐1 mentioned, “we're in a climb,” First Officer‐2 ignored the content, responding, “Damn it, where is [the captain]?”. Even when First Officer‐1 said, “I'm in [Take Off, Go Around],” First Officer‐2 continued to focus on the captain's return, saying, “Damn it, is he coming or not? We still have the engines! What the hell is happening? I don't understand what's happening.” First Officer‐2 failed to register the importance of First Officer‐1's statements, missing opportunities for correction.

Another example of ignoring is from US‐Bangla Airlines 211, where the pilot in command had a breakdown mid‐flight about a rumor that him and a colleague had sex in the cockpit. The first officer said “Sir, shouldn't I switch on the radar?” and the pilot ignored the query, saying, “That fucker Lamia, she made me… look at my fucking eyes… weeping… crying….” He did not acknowledge the radar‐related concern.

As the transcripts show behavior but not attitudes, it is challenging to determine whether ignoring resulted from not hearing, not valuing, or being unable to act on voice. For example, in Diamond N859PA, the pilot appeared to ignore multiple warnings that they were in restricted airspace. The ATC instructed, “You're entering a restricted flight area. You need to turn northeast bound to exit.” When N859PA did not respond, they repeated, “If you can hear this transmission, acknowledge.” With no response, the US Air Force intervened, stating, “You're entering restricted airspace and are being intercepted by an armed Air Defense fighter.” The N859PA pilot was on another frequency and could not hear or act upon the instructions.

###### 3.1.2.3.2 Token Listening

The final type of non‐engagement is token listening, where listeners verbally acknowledge safety voice, but their actions indicate little consideration of it. Token listening is rare, evident in inconsistencies between listeners’ verbal responses and their actions (e.g., saying they are climbing while descending the aircraft) and in subsequent conversation turns (e.g., voicers observing that the aircraft was descending).

For example, the ATC instructed South African DC‐40 to “Descend to [3,500’] now” and the cockpit responded, “Descending to [3,500’] now.” In the next turn, DC‐40's cockpit said, “We are now 4,000 ft descending,” indicating that despite confirming the instruction, they did not actually descend. The DC‐40 subsequently collided with another aircraft at 4000’.

Similarly, the ATC told Cessna N7022G to, “climb immediately, maintain 4,000’,” and the pilot verbally confirmed the request, saying, “4,000’, climbing immediately.” The ATC responded, “Okay, it looks like you're descending, sir. I need to make sure you are climbing, not descending,” highlighting the disconnect. The aircraft collided with mountains.

### 3.2 Conceptualizing How Safety Listening Behavior Prevents Harm

Having established that safety listening behaviors comprise (non‐)engagement with safety voice, we next examine how these behaviors prevent harm. We propose that engaging with safety voice helps avert accidents by fostering team situation awareness of problems and promoting the joint actions needed to address them. Situation awareness is team members’ understanding of dynamic environments and is crucial for safety in high‐risk contexts (Endsley 1995). Scholars suggest that situation awareness (and related concepts like team cognition) can be (un)shared between members (i.e., whether they are “on the same page”) and (in)accurate with respect to reality (Burtscher and Manser 2012). Ideally, teams have shared and accurate situation awareness; those lacking it are prone to errors, poor coordination, and failures (Salas et al. 2020).

In the transcripts, effective safety listening behaviors enhanced teams’ ability to prevent accidents by fostering shared and accurate situation awareness. Safety voice signaled information gaps that others were sometimes unaware of, revealing potential lapses in team situation awareness. Engaging with safety voice through action and sensemaking helped teams update their understanding of emerging issues. Conversely, non‐engagement prevented teams from correcting their understandings and addressing problems effectively.

Here, we describe how acting, sensemaking, and non‐engagement influenced situation awareness and accident prevention in the transcripts. For clarity, we only included relevant transcript lines.

#### 3.2.1 Acting

Safety listening as implementation prevented harm by incorporating voiced safety information into teams’ situation awareness, confirming their understandings, and executing suggested actions. For example, in KLM 1080/Transavia 5193, aircrews confirmed their understanding and agreement with the ATC's assessment of a collision course and took corrective action (Excerpt 1). Transavia 5193's cockpit not only confirmed the left turn instruction but also indicated awareness of the near miss by noting they could see the blue KLM aircraft.

**EXCERPT 1:** Safety listening as implementing leading to situation awareness (KLM 1080/Transavia 5193).

Line	Speaker	Transcript	Code	Interpretation
16	ATC	KLM 1080, turn right immediately	Safety voice	The ATC noticed that KLM1080 and TRA5193 were on a collision course and told KLM1080 to turn right
17	KLM1080	Right turn, KLM 1080	Implementing	KLM1080 turned right and confirmed
18	ATC	Transavia 5193, left turn immediately heading 180	Safety voice	The ATC told TRA5193 to turn left to ensure they do not collide
19	TRA5193	Left turn, got the KLM in sight—or the blue one—turning left to 180	Implementing	TRA5193 turned left and demonstrated understanding of the near collision by saying that they have the KLM in sight

*Note*: In 2018, KLM 1080 unexpectedly underwent a go‐around at Schiphol Airport, putting them into a mid‐air collision course with Transavia 5193 (“TRA5193”), which had just departed. Here, the ATC realized they were on a collision course and directed them to separate.

Abbreviation: ATC, air traffic controller.

Singapore 221 (Excerpt 2) also illustrates how safety listening incorporates concerns into teams’ situation awareness. The Singapore 221 cockpit told the ATC that they needed to abort landing twice due to the weather. The ATC listened and updated their awareness of Singapore 221's issues on both occasions. This communication aided its safe landing.

**EXCERPT 2:** Safety listening as implementing lading to team situation awareness (Singapore 221).

Line	Speaker	Transcript	Code	Interpretation
16	SIA221	Singapore 221, going around	Safety voice	SIA221 told the ATC they are aborting landing
17	ATC	Singapore 221, fly standard missed approach	Implementing	The ATC confirmed and told them to follow the standard procedure for missed approaches
23	SIA221	Tower, Singapore 221 super, established on ILS runway 07		SIA221 informed the ATC they were attempting the landing again on runway 7 using an instrument‐led approach
24	ATC	Singapore 221 super, g'day. Continue approach. Surface wind 040 degrees 18 knots		The ATC confirmed their approach and gave them the wind speed and direction
25	SIA221	Continue approach, Singapore 221		SIA221 confirmed
26	SIA221	Singapore 221, windshear—going around	Safety voice	SIA221 told the ATC they were aborting landing due to windshear
27	ATC	Singapore 221, acknowledge windshear, going around	Implementing	The ATC confirmed

*Note*: In 2016, inclement weather at Sydney Airport resulted in Singapore Flight 221 (“SIA221”) aborting landing (“going around”/missed approach) twice before successfully landing. This excerpt shows SIA221's two requests to go around and the ATC's confirmation both times.

Abbreviation: ATC, air traffic controller.

Declining also helped prevent accidents by fostering team situation awareness. When requests were declined, voicers updated their understanding of the listener's constraints and adjusted accordingly. For example, in US Airways 1549 (engine failure after bird strike; Excerpt 3), the flight crew declined a request to land at an airport due to their inability to comply. Instead, they chose to land in the river. Sharing limitations updated the team's situation awareness to reflect different perspectives of the emergency and its management.

**EXCERPT 3:** Declining safety voice updating team situation awareness (US Airways 1549).

Line	Speaker	Transcript	Code	Interpretation
222	Captain	Mayday mayday mayday. Uh this is uh Cactus 1539 hit birds, we've lost thrust (in/on) both engines we're turning back towards LaGuardia [Airport]	Safety voice	The captain told the ATC they hit birds, lost thrust in their engines, and that they were returning to the originating airport. The captain used the incorrect call sign
223	ATC	Ok uh, you need to return to LaGuardia? Turn left heading of uh 220	Implementing	The ATC confirmed intention to go to LaGuardia and gave instructions to do so
224	Captain	220		
231	ATC	Cactus 1529, if we can get it for you do you want to try to land runway 13?	Safety voice	The ATC asked if Cactus 1549 wanted to try runway 13 at LaGuardia, using the wrong callsign
233	Captain	We're unable. We may end up in the Hudson [River]	Declining	The captain said they were unable and warned they may land in the river
239	ATC	Arright Cactus 1549 it's gonna be left traffic for runway 31	Safety voice	The ATC suggested they land on runway 31 at LaGuardia
240	Captain	Unable	Declining	The captain said they were unable

*Note*: In 2009, US Airways Flight 1549 (“Cactus 1549”) struck birds shortly after takeoff from LaGuardia Airport. Here, the captain and the ATC were figuring out how to best land the aircraft, which had engine damage. The ATC suggested runway options, whereas the captain disagreed and said they will land in the Hudson River—which they eventually did.

Abbreviation: ATC, air traffic controller.

FedEx 1432 provides another example of declining that updated situation awareness. The FedEx cockpit was alerted about their collision course with Southwest 708 (Excerpt 4, line 12). The ATC redirected the voice, directing Southwest 708 to turn right (Excerpt 4, line 13), and Southwest 708 responded, “negative” (Excerpt 4, line 14). The ATC realized they were unable and revised their instructions for FedEx to climb and turn left (Excerpt 4, line 15).

**EXCERPT 4:** Declining safety voice updating team situation awareness (FedEx 1432/Southwest 708).

Line	Speaker	Transcript	Code	Interpretation
12	FDX1432	Southwest, abort! FedEx is on the go	Safety voice	FedEx 1432 asked Southwest 708 to abort takeoff because they were about to land on them. They did not specify which Southwest flight
13	ATC	Southwest 708, roger, you can turn right when able	Implementing	The ATC recognized that Southwest 708 was airborne and requested them to turn right
14	SWA708	Negative	Declining	Southwest 708 replied they could not turn right
15	ATC	FedEx 1432, climb and maintain 3000'. When able you can turn left heading 080	Safety voice	The ATC acknowledged Southwest's limitation and requested FedEx 1432 to climb and turn left
16	FDX1432	Turn 080, 3000'; FedEx 1432 heavy	Implementing	FedEx 1432 confirmed

*Note*: In 2023, FedEx 1432 (“FDX1432”) was landing on runway 18 L in foggy conditions, and the ATC had cleared Southwest 708 to take off from the same runway (18 L). Here, FDX1432 recognized and warned about the potential collision.

Abbreviation: ATC, air traffic controller.

Effective safety listening sometimes involved discerning between conflicting requests. For example, Japan Air Lines 907's crew faced a dilemma when the ATC mistakenly instructed them to descend, whereas their internal equipment advised them to ascend (Excerpt 5). The crew's decision to follow the incorrect ATC instructions and ignore the aircraft's alarm resulted in a shared but inaccurate situation awareness, nearly causing a mid‐air collision. This case demonstrates that listeners must navigate conflicting information to determine the best course of action.

**EXCERPT 5:** Safety listening following conflicting voice acts (Japan Air Lines 907/Japan Air Lines 958).

Line	Speaker	Transcript	Code	Interpretation
51	ATC	Japan Air 907, descend and maintain flight level 350, begin descent due to traffic	Safety voice (misguided)	The ATC told JAL907 to descend to avoid other planes despite intending this instruction to be for JAL958
52a	JAL907's RA	Climb climb climb	Dis‐confirmatory warning	JAL907's RA advised them to climb to avoid colliding with JAL958
52b	JAL907	Japan Air 907, descend and maintain flight level 350, traffic in sight	Implementing (misguided)	JAL907 confirmed descent and that they saw traffic. This action was misguided

*Note*: In 2001, two Japan Air Lines aircraft (“JAL907” and “JAL958”) narrowly avoided a mid‐air collision above Suruga Bay. Here, JAL907 received conflicting instructions to descend from the ATC and to climb from their resolution advisory system (RA; it informs cockpit crews of impending traffic collisions).

Abbreviation: ATC, air traffic controller.

#### 3.2.2 Sensemaking

Sensemaking following concerns typically developed shared and accurate situation awareness in two stages. First, teams recognized that safety concerns were raised, alerting them to potential hazards. Second, they explored these concerns, leading to a shared understanding of potential threats. For instance, in Lot Polish Airlines 5055 (Excerpt 6), safety listening triggered a sensemaking process. The team collectively understood the unfolding situation, ultimately identifying a fire and shutting down the engine.

**EXCERPT 6:** Sensemaking repairing situation awareness (Lot Polish Airlines 5055).

Line	Speaker	Transcript	Code	Interpretation
2	Crew	Is [there a] fire? What's going on?	Safety voice	This crew member asked others for help understanding the situation
3	Crew	Probably a fire	Elaborating	This crew member started to interpret the situation and communicated doubt with their evaluation (“probably”)
4	Crew	Engine? Shut it down!	Elaborating	This crew member correctly suggested it was an engine fire and requested the engine be shut down

*Note*: In 1987, after takeoff from Okęcie Airport, the crew applied maximum thrust on the engines, causing faulty bearings inside an engine to explode. Here, the engine had exploded, and the crews were addressing the situation. The fire rapidly spread, resulting in a total failure of all flight controls and the aircraft crashing.

Another example of sensemaking repairing situation awareness is Trans‐Air Service 671. Following an engine problem, the first officer suggested landing at a military airfield, but the captain disagreed, thinking the runway was too short (Excerpt 7, lines 39–41). The first officer inquired about the runway length, and the ATC confirmed it was 4000 m. This information led the captain to agree that the runway was sufficient, ensuring a coordinated decision.

**EXCERPT 7:** Sensemaking leading to situation awareness (Trans‐Air Service 671).

Line	Speaker	Transcript	Code	Interpretation
39	First officer	Can we land there?	Safety voice	The first officer suggested landing at the military airfield
40	Captain	No	Declining	
41	Captain	Too short, too short!	Declining	The captain believed that Istres’ runway was too short to safely land on
42	First officer	How long is the runway on this military airfield?	Questioning	The first officer acknowledged the captain's concern by verifying the runway's length with the ATC
43	ATC	Four thousand meters	Elaborating	
44	Captain	Oh yeah, OK	Implementing	

*Note*: In 1999, Trans‐Air Service 671 was a cargo flight that encountered an engine separation en route and safely diverted to Istres military airfield. Here, the first officer suggested landing at Istres and corrected the captain's misperception that the runway was too short.

Abbreviation: ATC, air traffic controller.

Teams sometimes created risk by acting when sensemaking was required. For example, in Air Canada 759, the aircraft mistakenly aligned to land on a taxiway with four commercial aircraft. The cockpit hinted, “we see some lights on the runway there. Please, confirm we are cleared to land?” (Table 5, line 4). The ATC confirmed clearance for runway 28R, neglecting the concern about the lights. This lack of sensemaking led to a missed opportunity to recognize the aircraft was heading toward the taxiway, increasing risk.

Teams also increased risk when their sensemaking created shared but inaccurate situation awareness (i.e., developing a collective yet erroneous understanding). In Air New Zealand 901, the team was flying in poor visibility and the first officer inquired about their position relative to a mountain (Excerpt 8, line 57). The guide incorrectly stated it was to their left (Excerpt 8, line 58). The team, through sensemaking, mistakenly concluded they were flying over McMurdo Sound, despite being on a collision course with Mount Erebus.

**EXCERPT 8:** Sensemaking resulting in shared yet inaccurate team situation awareness (Air New Zealand 901).

Line	Speaker	Transcript	Code	Interpretation
57	First officer	Where's Erebus in relation to us a[t] the moment	Safety voice	The first officer asked where the plane was in relation to the mountain
58	Guide	Left, about 20 or 25 miles	Elaborating (misguided)	The guide wrongly said that the mountain was left of them rather than ahead
59	First officer	Yep, yep	Implementing (misguided)	
60	Flight engineer	I'm just thinking of any high ground in the area, that's all	Elaborating	The flight engineer was looking for landmarks to situate them
61	Guide	I think it'll be left	Elaborating (misguided)	The guide wrongly repeated that the mountain was to the left
62	Flight engineer	Yes, I reckon about here	Elaborating (misguided)	The flight engineer agreed

*Note*: In 1979, Air New Zealand 901 left Auckland International Airport for a sightseeing flight to Antarctica with cloud cover obscuring terrain. Moreover, the crew encountered a series of errors in navigation coordinates that led to the aircraft deviating from its planned flight path. Here, the crews were figuring out where they were in relation to Mount Erebus and wrongly believed that they were flying over McMurdo Sound instead of on a collision course with Mount Erebus.

#### 3.2.3 Not Engaging

Responses that failed to engage with safety voice prevented shared and accurate situation awareness. For instance, in Kalitta 808, the flight engineer raised valid concerns about stalling (Excerpt 9, line 7); however, the captain and first officer dismissed these warnings while searching for the runway. The aircraft stalled and crashed.

**EXCERPT 9:** Ignoring safety voice preventing situation awareness repair (Kalitta 808).

Line	Speaker	Transcript	Code	Interpretation
7	Flight engineer	You know, we're not gettin' our airspeed back there	Safety voice	The flight engineer hinted that the aircraft was stalling
8	Captain	Where's the strobe?	Dismissing	The captain did not register this concern because they were focused on identifying the strobe light
9	First officer	Right down there	Dismissing	
10	Captain	I still don't see it	Dismissing	

*Note*: In 1993, Kalitta 808 was attempting to land at the US Naval Station Guantanamo Bay in Cuba. Here, the flight engineer raised concerns about stalling; however, the captain and first officer ignored these concerns, focusing on finding the strobe light indicating the landing runway.

After initial non‐engagement, voicers or others could escalate or amplify their voice acts to prompt engagement. Escalating voice involves the same voicer repeating their concern, often with increased urgency, whereas amplifying voice involves a third party reiterating the safety message. These additional voice acts are typically more direct and explicit than the initial concerns.

For example, the ATC engaged in escalating voice by repeatedly instructing Air China 428 to turn right after receiving no response to their initial warnings (Excerpt 10, line 19). This escalation helped the crew understand the imminent collision risk, leading to situation awareness and corrective action.

**EXCERPT 10:** Escalating safety voice after non‐engagement (Air China 428).

Line	Speaker	Transcript	Code	Interpretation
16	ATC	Air China 428, turn right immediately. Turn right immediately. Heading 0—correction—heading 270. Terrain ahead. Expedite climb	Safety voice	The ATC told CCA428 to turn right and climb immediately as there was terrain ahead
17	ATC	Air China 428?		No response from CCA428; the ATC checked if they have heard
18	CCA428	[Radio noises]		Unclear response from CCA428
19	ATC	Air China 428, expedite climb. Terrain ahead—terrain alert! Expedite passing 5000 feet. Expedite!	Escalating safety voice	
20	CCA428	Expedite, Air China 428	Implementing	

*Note*: In 2017, Air China 428 (“CCA428”) incorrectly turned left heading toward mountains upon departure from Hong Kong International Airport. Here, the ATC instructed CCA428 to turn right immediately and expedite climb, averting an accident.

Abbreviation: ATC, air traffic controller.

Amplifying voice helped prevent potential accidents by reinforcing concerns and prompting action after initial non‐engagement. For example, United 1448 mistakenly taxied onto an active runway and reported this to the ATC, but the ATC initially ignored the message and dismissed further reports, even clearing other aircraft for takeoff (Excerpt 11). US Airways 2998 amplified United 1448's concerns by refusing to take off until the situation was clarified. This persistent refusal forced the ATC to investigate United 1448's position, ultimately preventing harm.

**EXCERPT 11:** Moving from non‐engagement to investigation (United Airlines 1448).

Line	Speaker	Transcript	Code	Interpretation
108	UAL1448	And uh United 1448 we're approaching kilo here uh um—somebody just took off	Safety voice	Muted voice signaling that UAL1448 was in the wrong place, which might have been a runway
109	ATC	United 1448 you shouldn't be anywhere near kilo hold your position please just stop	Implementing	The ATC did not realize that UAL1448 was on an active runway and requested them to hold position
In these 18 lines, UAL1448 escalated their voice act three times and was ignored and told to “standby” by the ATC, who cleared FedEx 1662 for takeoff. FedEx 1662 took off and passed close overhead to UAL 1448, which was at the end of the runway. The ATC cleared USA2998 for takeoff and USA2298 amplified UAL1448's voice by refusing to take off, whereas UAL1448's position was unknown				
127	UAL1448	Ma'am this is United 1448 we're on 23R we're looking at kilo straight ahead if we can go straight we can get on kilo and get off the runway	Escalating safety voice	UAL1448 told the ATC that they were on runway 23R and suggested again that they could exit the runway via taxiway kilo
128	ATC	United 1448 standby please don't talk I have other things I need to do	Dismissing	The ATC told UAL1448 not to speak again
130	ATC	US Air 2998 runway 5R fly runway heading cleared for takeoff	Dismissing	The ATC cleared USA2998 for takeoff despite not knowing UAL1448's position for a second time
131	USA2998	Uh tower US Air 2998 till we figure out what's going on down there we're just going to stay clear of all runways	Amplifying safety voice	USA2998 restated their concern with taking off, whereas UAL1448's position was unknown
132	ATC	US Air 2998 roger hold short of runway 5R he's not anywhere near the runway but you can hold short	Implementing	The ATC acknowledged USA2998's concern
In these 11 lines, the ATC gave instructions to other uninvolved aircraft, noting that “Tower US Air 2998 isn't going yet because I have a United who doesn't know where the hell he is” (line 138). She said she intended to move UAL1448 so that USA2998 would takeoff				
143	ATC	United 1448 understand you're on runway 23R have you gone past taxiway kilo and tango one?	Questioning	The ATC engaged with UAL1448 asking for information on their position
144	UAL1448	Ma'am we're on 23L and 16 and I am facing into kilo at this point the nose is uh just over 23L	Elaborating	UAL1448 provided information on their whereabouts. UAL1448 and the ATC engaged in sensemaking until they understood UAL1448's position and moved the aircraft

*Note*: In 1999, United Airlines 1448 (“UAL1448”) failed to follow its taxi clearance after landing at night in thick fog. It mistakenly ended up at the edge of the runway it had just landed on. The ATC—who did not have surface radar available—made incorrect assumptions about UAL1448's position and cleared US Airways 2998 (“USA2298”) for takeoff despite that runway being obstructed.

Abbreviation: ATC, air traffic controller.

After non‐engagement, situation awareness was repaired when listeners acted upon additional voice acts. Escalating and amplifying voice arose from voicers’ and third parties’ belief that listeners did not engage with their concerns and the team's situation awareness remained fractured. Escalating and amplifying illustrate that safety communications are iterative and multi‐turn interactions (i.e., not one‐shot) and demonstrate that persistence may solicit effective safety listening behaviors.

## 4 Discussion

We explored how safety listening behaviors manifest and prevent accidents in high‐risk contexts. Our analysis of flightdeck transcripts identified effective listening as engagement with safety voice, comprising action (implementing and declining) and sensemaking (questioning and elaborating). Non‐engagement—characterized by dismissing and token listening—often contributed to accidents.

We theorized that safety listening behaviors avert harm by fostering shared and accurate situation awareness. Through action and sensemaking, teams integrated safety voice's content into their understanding of evolving risks and adapted their behavior to address problems. Conversely, non‐engagement with voice impeded teams’ situation awareness repair, impeding coordination and problem‐solving.

### 4.1 Theoretical and Practical Considerations

We make several contributions to the literature. First, we advance safety listening research by explaining its behavioral rather than attitudinal nature. Scholars often operationalize listening as voicers’ perceptions (e.g., feeling heard), listeners’ attitudes (e.g., openness to feedback), and listeners’ behaviors (e.g., nodding; Kluger and Itzchakov 2022; Yip and Fisher 2022). Conversely, we argue that safety listening behavior is observable engagement with safety voice (Pandolfo et al. 2025). In high‐risk situations, listeners’ behavioral responses—rather than perceptions and attitudes—are how voiced requests translate into accident prevention. For example, listeners disconfirming misguided voice might appear like poor listening; however, the disagreement is a considered rejection of incorrect information.

Second, we specify that shared and accurate team situation awareness is the mechanism through which safety listening behavior prevents accidents. Current safety communication research lacks a detailed understanding of the psychological mechanisms underlying how voice and listening prevent harm. Likewise, although the situation awareness literature identifies teams’ information sharing as an antecedent to situation awareness development (Parush et al. 2011), it has not explored these conversations’ pragmatics. Here, we integrate these literatures to suggest that in dynamic situations, individuals speak up to improve team situation awareness and ensure problem recognition. Safety listening, therefore, updates team situation awareness, allowing teams to remain aware of evolving situations and effectively address hazards. Rather than one‐shot communications, safety listening is an ongoing process involving multiple conversation turns to build and maintain situation awareness. Safety listening behaviors’ effectiveness therefore depends on whether they create a shared and accurate understanding of risks. As illustrated by the transcripts, this model applies to bounded teams (e.g., cabin crew), extended teams (e.g., ATCs), and across organizations (e.g., air crew and safety departments on technical risks).

Third, we demonstrate how researchers can use behavioral trace data to assess naturalistic safety communications. Our framework classifies safety listening behaviors in aviation, answering calls for using ecologically valid data and behavioral measures in psychology (Baumeister et al. 2007). Future research may adapt this framework for other high‐risk industries (e.g., healthcare), in situ observations, or to train large language models (e.g., ChatGPT) to quickly identify safety listening behaviors in sizeable datasets.

Finally, our identification of safety listening's behavioral markers can inform interventions to improve organizational safety communications. At present, interventions focus on improving listeners’ attitudes (e.g., suspending judgment; Itzchakov 2020), encouraging more assertive voice (Jones et al. 2021), and improving psychological safety (O'Donovan and McAuliffe 2020). While valuable, these interventions may not translate into developing safety listening skills (e.g., distinguishing between conflicting voice acts) or identifying the best listening behaviors in different contexts. The behaviors identified here can form behavioral markers for assessing listening in live scenarios and tools to provide structured feedback to improve listening skills. Likewise, trainings can incorporate real conversation transcripts to demonstrate effective safety listening behaviors (Stokoe 2014).

### 4.2 Implications for the Safety Voice, Safety Listening, and Situation Awareness Literatures

Our findings uncover nuances and challenges to existing safety communication and accident prevention models. First, although researchers typically view voicers and listeners as dyads (Yip and Fisher 2022), we found that multiple voicers can be involved, including other team members and third parties (e.g., United 1 in the Air Canada 759 case in Table 5). As individuals may offer contradictory safety information, investigation is necessary to determine which voice to engage with; amplifying voice may aid this decision (Bain et al. 2021; Satterstrom et al. 2024).

Second, scholars often treat safety voice and listening as one‐shot rather than as part of an iterative and patterned conversational process. Our transcripts demonstrate multiple turns of voice and listening, including additional voice acts that revealed shared misperceptions among team members. Voicers sometimes became listeners in later conversation turns, and vice versa, indicating that interlocutors’ roles were fluid. Presenting communication as voice‐listening‐outcome likely stems from surveys assessing listening in the average (e.g., whether voicers believe that others generally listen; Tucker and Turner 2015). Safety communication's iterative nature should be conceptualized in safety climate and culture models (e.g., Reader et al. 2015), emphasizing how organizations foster safety listening.

Third, safety voice can be misguided (Hald 2023). In such instances, effective safety listening behaviors engaged with voice but rejected inaccurate concerns and avoided forming incorrect situation awareness. One possible listening skill is navigating a “signal to noise ratio” where listeners must consider concerns, discerning between those which are legitimate (“signal”) and erroneous (“noise”). Scholars predominantly investigate listening, which confirms to voice acts (e.g., agreement), assuming that all concerns are informed and legitimate (e.g., Barlow et al. 2023). Yet, rejecting misguided voice maintains safety and situation awareness and may be an important teaching moment. Likewise, it may be easier for listeners to engage with voice, which aligns with their understanding (e.g., confirmation bias; Klayman 1995), than with surprising messages; future research can test this.

Fourth, although scholars typically view no response to safety voice as ignoring (Noort et al. 2021b), we suggest that listeners do not always hear or understand voice acts. For instance, voicers may be on different radio frequencies or mumble their messages. If voicers suspect that listeners did not hear their message, they may engage in escalating voice, clearly repeating their concern until they believe they are heard. Hearing failures can also occur due to human errors (e.g., sending complaints to the wrong address), technological issues (e.g., broken radio), confusing reporting systems, or environmental obstructions (e.g., voice muffled by gunfire; Wilson et al. 2007). Future research should explore hearing as a mediator between safety voice and listening, incorporating it into theoretical models.

### 4.3 Limitations

This research has limitations. First, we examined safety communications preceding incidents; we did not examine communications that were appropriately addressed during otherwise normal flights. As transcripts were only available for flights resulting in an incident, we could not explore how safety communications were delivered and addressed in status quo flights. For instance, misguided speaking up and listening as declining may occur more frequently when first officers in training are flying with more experienced captains. Future research should examine base rates of safety voice, safety listening, and misguided communications in normal operations, perhaps using transcripts of instructional flights.

Second, when operationalizing safety voice, the distinction between raising concerns and routine instructions was sometimes blurred. Although Noort et al. (2021b, 2021c) delineated safety voice, safety silence, and routine communications based on whether the voicer was concerned, we sometimes found it challenging to identify concern from the transcripts alone. As such, like Barton and Sutcliffe (2009), we positioned voice as instructions aiming to disrupt behavior and realign understandings of the situation. Future research should analyze incidents where survivors’ post hoc interview transcripts are publicly available, examining their in situ conversations, their recalled thoughts and behaviors, and the official incident reports.

Third, near misses were ATC recordings, whereas crashes were predominantly CVR. CVRs are typically overwritten after near misses (Kelleher 2023) and are unavailable for recent crashes. Ideally, we would have exclusively used CVRs because they are comprehensive; however, using ATC recordings broadened our scope to include near misses and recent accidents.

Fourth, despite our random sampling, 14 out of 30 incidents involved American aircraft or occurred in America. Many ATC recordings are from American airports (*LiveATC.Net* 2025), and over half (*n* = 87/170) of Noort et al. (2021a)’s transcripts involved American aircraft or airspace. In some countries (e.g., the United Kingdom), the public are not permitted to record ATC feeds; consequently, their airports have limited available recordings. To test our findings’ validity, we supplemented the 30 transcripts with an additional 15; however, these were also skewed toward America.

Last, we describe safety listening behaviors in aviation conversations. Although our findings may generalize (e.g., Weick and Sutcliffe 2003 studied sensemaking in healthcare), other contexts may have nuances not captured in aviation (e.g., system dynamics). Future research should measure naturalistic safety listening behaviors in non‐aviation contexts (e.g., police interviews), as leading indicators (i.e., in situ, routine monitoring), and in written formats (e.g., emails).

## 5 Conclusion

Safety listening is defined in terms of listeners’ behavioral responses to safety voice to prevent harm; however, limited research describes safety listening behaviors in high‐risk contexts and conceptualizes their relationship with accidents. We analyzed flightdeck conversations preceding incidents, finding that effective safety listening behaviors engage with safety voice (i.e., acting and sensemaking) and safety listening repairs team situation awareness, leading to accident aversion. Our contribution lies in establishing the behavioral nature of safety listening and demonstrating that engagement with safety voice offers an accurate and practical explanation of how safety listening shapes organizational safety outcomes.

## Ethics Statement

This research received ethical approval from the London School of Economics (Ref. 90926).

## Data Availability

The data that support the findings of this study are openly available in Open Science Framework at https://osf.io/nm7hz/?view_only=0b1a22ab52384728baa24d4c51b8e4af.

## The deductive coding framework

**Table:**

Code	Description	Relevant terminologies	Example
Response consistent with voice	Speech and/or action consistent with the voice request	Immediate action, verbally affirmed (Noort et al. 2021b); content is implemented, short approval, elaboration (Lemke et al. 2021); accepted (Reader 2022); sensemaking (Weick 1988, 1990)	*Air Canada 759* United 1: United 1, Air Canada flew directly over us ATC: Yeah, I saw that, guys
Response inconsistent with voice	Speech and/or action inconsistent with the voice request	Disaffirmed (Noort et al. 2021b); denied (Reader 2022); rejection (Lemke et al. 2021); avoiding, delegitimizing, limiting tactics (Gillespie 2020); avoiding, opposing (Satterstrom et al. 2021)	*Horizon Air* ATC: We're just trying to find a place for you to land safely Hijacker: Yeah, I'm not quite ready to bring it [aircraft] down yet
No response	No verbal or behavioral response following voice	Ignored (Noort et al. 2021b; Reader 2022); inaction (Reader 2022); no verbal or non‐verbal reaction, content not implemented (Lemke et al. 2021); avoiding tactics (Gillespie 2020); avoiding (Satterstrom et al. 2021)	*Diamond N859PA* ATC: N859PA, this is Philly Approach. If you're on frequency, acknowledge. You're entering a restricted flight area. You need to turn northeast bound to exit ATC: N859PA, this is Philly Approach on Guard. If you can hear this transmission, acknowledge
